# Decomposing gender inequalities in self-assessed health status in Liberia

**DOI:** 10.1080/16549716.2019.1603515

**Published:** 2019-06-03

**Authors:** Conrad Murendo, Gamuchirai Murenje

**Affiliations:** Monitoring, Evaluation, Impact and Learning, International Crops Research Institute for the Semi-Arid Tropics, Bulawayo, Zimbabwe

**Keywords:** Gender and Health Inequality, Gender inequality, health, non-linear, decomposition, Liberia

## Abstract

**Background**: Understanding the magnitude of inequalities and drivers for reducing gender-related health inequalities is crucial in developing countries. This is particularly the case for Liberia with its very high level of gender-related inequalities in health and health outcomes.

**Objective**: This paper assesses the magnitude of gender health inequalities and the relative contribution of different factors to health inequality in Liberia.

**Methods**: Data came from the Liberian Household Income Expenditure Survey 2014. A two stage sampling methodology was used and 4,104 households were randomly selected and interviewed. The main variable of interest is dichotomised, good versus poor self-assessed health. Gender-related health inequality is assessed using the Oaxaca–Blinder decomposition for non-linear models. The decomposition reveals the magnitude of inequality and contributions of different factors.

**Results**: We found large gender disparities (0.054, p < 0.01) characterised by women disadvantages in health status. In addition, the gender health disparities are mostly pronounced in rural areas. About 54% of the gender inequalities in health status were explained by the differences in endowments. Equalizing access to information, wealth and utilization of mosquito nets would reduce the gender gaps by 44, 5 and 4%, respectively.

**Conclusions**: Addressing gender health inequalities inter alia requires access to health information (i.e. electronic and print media), gender responsive interventions that improve wealth in key sectors (i.e. education, employment, social protection, housing, and other appropriate infrastructure). In addition, the government, private sector and civil society should ensure that the health sector provides access to quality mosquito nets and improved health services including preventive care in order to reduce disease burden.

## Background

Understanding the nature and magnitude of the differences in health experiences between men and women is crucial to identify the processes generating ill-health for everyone and addresses imbalances in the diagnosis of disease and subsequent treatments which tend to favour men and disadvantage women [1,2]. Self-assessed health (SAH) is one of the widely used measure based on a person’s self-assessment of his/her status and is a remarkably reliable measure that is consistent with the actual health status of the respondents [3,4]. The SAH is a more preferred measure over individual disease conditions such as diabetes, tuberculosis, and injuries or other self-reported illnesses, because it is multidimensional and includes inter alia physical, functional, coping, and well-being aspects [3,5,6]. Furthermore, SAH is validated as a predictor of mortality and morbidity [3,7] and is strongly associated with declared chronic illnesses [8].

Empirical research on gender differences in SAH has yielded mixed findings. Many studies suggest that women report worse self-assessed health than men [4,9,10], while others report no gender differences at all [11,12] and socio-economic status contributes significantly to the differences [4]. Onadja et al. [9] found that women reported worse cognitive impairment and mobility disability than men and that nutritional status and education opportunities reduced the gender differences in Burkina Faso. In their study, Miszkurka et al. [13] found that women had higher odds of mobility disability than men at every age group in Burkina Faso, Mali and Senegal. In India, Singh et al. [10] highlights that women reported worse cognitive health than men and that education, marital status, caste, religion, tobacco consumption and chronic health status contributed to the reduction in gender gap. Differential gender access to education and income opportunities [14,15] tend to disadvantage women in some societies and exacerbate the gender differences in health. Education is associated with many factors that have a significant impact on health-seeking behaviour, reproductive behaviour, use of contraception, and maternal and child health and nutrition [16,17]. According to [17], higher levels of education are positively associated with better employment opportunities and higher income, and can provide the knowledge and skills necessary to access good-quality health services. In turn, higher levels of income and wealth are associated with better health status, access to healthy food, health care and housing [17,18]. On another note, the use of solid fuels for cooking and heating can lead to high levels of indoor smoke, a complex mix of health-damaging pollutants that could increase the risk of contracting diseases [19] especially for women who do most of the household chores in developing countries.

Despite the important role of gender in health, empirical studies addressing the gender difference in SAH in Liberia are scarce. Gender-based health inequalities can be resolved only by knowing whether and where any such inequality exists. In designing health interventions in Liberia, it is essential to understand factors that drive health differentials between men and women. This is very crucial given that gender inequality in social and economic domains has been the subject of national debate in Liberia which ranks 143 out of 147 countries in the Gender Equality Index and the literacy level is about 62% for men and 33% women [20,21].

This paper differs from earlier studies in a number of respects. First, we analyse the gender disparities in health status using recent survey data. Second, we use the SAH, a multidimensional and more encompassing measure of health [3,5]. Third, with the exception of [22–24] most of the empirical studies have not measured the degree to which the covariates contribute to disparities. While gender differences in the distribution of health risk factors might contribute to gender inequalities in health status, it is also possible that these characteristics have differential effects on health risk for men and women. For example, women are physiologically and culturally more vulnerable to sexually transmitted infections than men [25,26]. Therefore, gender inequalities in health may arise even in the absence of differences in health risk behaviour. Similarly, the effects of socio-economic characteristics, like education on health status may be different for men and women due to socio-cultural factors including discrimination against women in education and labour market. It is indeed important to specifically quantify the magnitude of the inequalities and the degree to which the covariates contribute to the health inequality [27] as we do in this paper. Even in the presence of an interaction term, the simple regression model fails to quantify the degree to which the covariates contribute to the health inequality.

In this study, we used an extension of the Oaxaca–Blinder decomposition for non-linear models to investigate the relative contributions of variations in the distributions of health status versus their differential effects in producing gender inequalities in health status in Liberia [27,28]. Again, the recent few studies that have analysed the contribution of covariates to the health inequalities are mostly concentrated in developed countries [22,23] and little is known in developing countries [24]. This knowledge is crucial for designing more effective health policies and programmes in developing countries. For example, if gender inequalities in health are explained mainly by the distribution of socio-economic characteristics by gender, then programmes that reduce gender differences in socio-economic resources might mitigate gender inequalities in health [25]. However, if health gender disparities are primarily due to men and women’s differential ability to use similar resources to alter their health status, then programmes that focus solely on equalizing resources may not achieve their objectives and may even exacerbate health differences by gender [25].

## Gender and health in Liberia

Liberia is one of many developing countries in Africa, experiencing rapid epidemiological transition, which involves dealing with both communicable and non-communicable diseases at the same time [29,30]. According to Parnarouskis et al. [29] much of Liberia’s population remain in severe poverty, which disproportionally affect women due to unequal access to resources and employment opportunities and this continues to create disparities in health. Furthermore, the harmful traditional practices including son preference, early marriages, female genital mutilation, gender-based violence and overload of family care work, increase the risk of ill health among women than men [20]. Women in Liberia have less access to education, health care, property, and justice when compared to men. This is partly attributed to poor investment in health care and education for women, and their poor access to services, as well as legal and cultural barriers that restrict women’s decisions [20]. In 2010, about 89% of women in the labour force were in vulnerable employment compared to 69% of men in the labour force. More recent data found that the female infant mortality rate in Liberia (48 deaths/1000 live births) is lower than the male infant mortality rate of 58 deaths per 1000 live births [21]. Female mortality in Liberia is also increased by the prevalence of female genital mutilation which affects more than two-thirds of women and girls. Despite this, Liberian women enjoy a higher life expectancy at birth (66 years) than men (61 years) [21,30].

## Methodology

### Data

The data for this article are drawn from the Liberian Household Income Expenditure Survey 2014 (HIES 2014) that was administered to a representative sample of households. The survey used a two stage sampling methodology. At the first stage 409 enumeration areas were selected. At the second stage 4,104 households were randomly selected and interviewed [31]. The HIES 2014 collected detailed information at household level on the following topics: education, health, employment, water and sanitary practices, household resources, grants, crime, conflicts and recent shocks to household wealth.

### Measures

#### Dependent variable

Self-assessed health status (SAH) is used as the dependent variable in our study. In the Liberia dataset, respondents rated their health status in one of the following seven categories: very satisfied, satisfied, somewhat satisfied, neither satisfied nor dissatisfied, somewhat dissatisfied, dissatisfied, very dissatisfied. This was further classified to obtain ‘good health’ (i.e. corresponding to very satisfied and satisfied).

#### Independent variables

The choice of independent variables used in the analysis and associated with the gender differences in SAH were guided by literature [3,4,8,32], and included socio-economic characteristics (e.g. age, marital status, household size and education), wealth, housing, water and sanitation, fuel types, information access, mosquito nets and illness in the last 30 days. Location was measured by whether a household lived in urban or rural area. Education of household head was measured by the question: ‘Did you ever go to primary or secondary or university and above?’, while literacy was measured by whether household head can read and write. Marital status question asked: ‘What is your marital status and responses included monogamous married, polygamous married, living together, separated, divorced, widow(er)?’ We created a binary variable of currently married versus others.

Per capita expenditure is computed by dividing the total household food and non-food consumption expenditure by household size. The type of material used for flooring is also an indicator of socioeconomic status and to some extent determines the household’s vulnerability to disease-causing agents [33]. Concrete or cement floors were classified as clean and durable while earthen floors (made of earth, sand, or mud) where denoted unclean. The source of drinking water is important because waterborne diseases, including diarrhoea, typhoid and cholera, are prevalent in developing countries including Liberia [32]. The sources of water in the rainy and dry seasons were dichotomised to obtain ‘clean water’ (i.e. corresponding to piped water, hand pumps/protected wells, and protected springs expected to be relatively free of the agents responsible for these diseases). Other sources such as unprotected wells, rainwater, rivers or streams, and ponds, lakes, or dams are more likely to carry disease-causing agents and these were coded as 'unclean'.

Toilet facility was measured by the question: ‘What is the main toilet facilities usually used in this household?’ The answers included flush for household only, flush and shared, covered pit latrine, open pit latrine, ventilated pit latrine, bush and others. A household is classified as having an improved and hygienic toilet if the toilet flushes into a piped sewer system, septic tank, or pit latrine; ventilated improved pit latrines; and pit latrines with a slab which separates the waste from human contact [32]. Lighting fuel was measured by the question: ‘What is the household’s major fuel used for lighting?’ Electricity, Jacko and Chinese lamps were classified as clean sources while wood, kerosene lamp and candle were denoted unclean. Solid fuels including firewood and coal were classified as unclean and electricity and gas were classified as clean. Access to information was measured by whether a household listened to current news on the radio frequently in the past 12 months. The use of mosquito nets, an important malaria prevention technique [34] was classified as one if the head slept under the mosquito net yesterday and zero otherwise. Following Ataguba et al. [3], we measured household illness as the self-reported illness or injury in the past 30 days.

### Statistical analysis

We begin our analysis by comparing measures of health status as well as other explanatory variables between men and women. Our study focus on the male and female gap in SAH among household heads. However, to be consistent with majority of gender studies, we refer this as the men-women gap in SAH in the whole of the article. We use the Student’s *t*-test to examine whether the socio-economic differences in men and women are statistically significant. We then conduct the Oaxaca-Blinder decomposition techniques to explain the gender health disparities in Liberia. The Oaxaca-Blinder decomposition method divides the health disparities between men and women into a part that can be explained by differences in the levels of observed covariates such as socio-economic and demographic characteristics controlled in the regression, and a residual part that cannot be accounted for by any observed differences in the covariates [27,28,35].

The decomposition is done using the recent extension of the Oaxaca-Blinder method developed by Powers et al. [27] for non-linear dependent variables. The non-linear logit Oaxaca-Blinder decomposition in the context of the men-women disparities in health status can be written as:

$$\begin{array}{rl} & {\overset{ˉ}{y}}^{M}-{\overset{ˉ}{y}}^{R}=\left[F\left(X^{\overset{ˉ}{M}}{\hat{\beta }}^{M}\right)-F\left(X^{\overset{ˉ}{R}}{\hat{\beta }}^{M}\right)\right]\\ & \;\;\;\;\;\;\;\;\;\;\;\;\;\;\;\;\;\;\;\;\;\;\;+\left[F\left(X^{\overset{ˉ}{R}}{\hat{\beta }}^{M}\right)-F\left(X^{\overset{ˉ}{R}}{\hat{\beta }}^{R}\right)\right] \end{array}$$

Where the superscripts M and R stand for the men and women groups, $\overset{ˉ}{y}$ is the outcome of interest averaged over each group, and $\hat{\beta }$ are estimated by logit model, F() is the cumulative distribution function of the logistic distribution. Note that X and β in this expression are in a vector form. The first square parenthesis measures the disparity due to the differences in characteristics (‘characteristic effect’) and the second parenthesis measures the disparity due to the different effects of the observed characteristics (‘coefficient effect’). A positive coefficient in the explained components (distributions of characteristics) indicates the expected reduction in health inequality if men were equal to women on the distribution of covariates [27]. Due to the nonlinearity of the factors, the detailed decomposition is not as straightforward as the ‘linear’ decomposition.

The literature has developed different approaches [27,35]. This approach replaces each covariate of one group with that of the other group to quantify the contribution of each covariate. However, the problem with this approach is that the result changes with the order of the replacement. Our study takes a different method that overcomes various problems with ordering the variables entered into the decomposition (i.e. the problem of path dependence) and the sensitivity of the results of the coefficient portion of the decomposition to the choice of the reference category when regression models include dummy variables (i.e. identification problem) [27,36]. All STATA commands for the decomposition analysis have already been published elsewhere [27]. For robustness, we checked how the regression model is sensitive to the exclusion of the wealth variable – per capita expenditure, given that the collection of expenditure data is subject to considerable measurement error in developing countries [37].

## Results

### Descriptive results

Table 1 provides the summary statistics for all variables used in this study. The results show significant women disadvantages in health status, education, wealth, access to information and mosquito nets. A higher proportion of men sleep under a mosquito net compared to women. A higher proportion of women reported illness in the last 30 days compared to men. The descriptive results also show that the significant women disadvantages in health status, education, wealth, access to information and mosquito net exists in rural areas.

**Table 1.** Overall and rural sample characteristics.

	Overall sample			Rural sample		
Variable	Men	Women	Differences	Men	Women	Differences
SAH	42.3	37.0	−5.0***	39.6	29.9	−10.0***
Urban	34.5	46.5	12.0***	-	-	-
Age	46.5	45.7	−0.70	47.2	48.7	1.5**
Married	66.2	20.4	−46.0***	72.3	24.6	−48.0***
Household size	4.64	3.84	−0.80***	4.6	3.9	−0.7***
Education	66.6	34.5	−32.0***	59.6	21.1	−38***
Literate	61.9	31.6	−30.0***	53.9	18.2	−36***
Expenditure	53,968.2	48,416.0	−5552.20***	46,665.1	38,988.3	−7676.9***
Roof	64.9	72.4	8.0***	51.1	57.1	6.0***
Floor	41.6	47.1	6.0***	22.7	24.6	2.0
Toilet	26.5	27.5	1.0	17.6	16.4	−1.0
Lighting fuel	61.8	61.5	−0.3	54.5	52.5	−2.0
Cooking fuel	29.0	36.5	7.0***	8.9	10.3	1.0
Clean water rainy	61.9	71.8	10.0***	49.5	58.2	9.0***
Clean water dry	59.0	69.1	10.0***	45.7	55.2	9.0***
Radio	61.2	29.2	−32.0***	56.7	22.9	−34.0***
Mosquito net	51.5	48.0	−3.0**	54.5	51.5	−3.0
Illness	25.8	37.4	12.0***	25.4	36.4	11.0***
*N*	3017	1087		1976	582	

Note: 1 USD = 133 Liberian dollar as of May 2018. *, **, ***. Statistically significant at the 10%, 5%, and 1% level, respectively based on two-tailed t-test. Mean and percentages are shown for continuous and dummy variables respectively.

### Econometric results

Table 2 shows the contribution of individual characteristics to gender inequality in health status. Results show significant gender gap in health status (0.054, p < 0.01). About 54% of the gender inequalities in health status were explained by the differences in distributions of characteristics (endowments) between men and women. The gender inequalities in health status would be eliminated if men and women had similar levels of access to information, wealth, mosquito nets and illness. For example, if men and women had the same distribution of access to information, the gender inequality in health status would be reduced by 44%. We do find that illness account for nearly 14% of the explained gap in gender differential. Equalizing wealth and utilization of mosquito nets would reduce the gender gap by 5 and 4% respectively.

**Table 2.** Logit decomposition of men-women gap in health status.

	Estimate	Standard error	Percent
Raw difference	0.054***	0.016	100
Explained: due to difference in characteristics	0.030***	0.012	54.4
Unexplained: due to difference in coefficients	0.025	0.020	45.6
**Due to difference in characteristics**			
Urban	−0.001	0.003	−1.7
Age	−0.005***	0.000	−8.5
Married	0.003	0.009	5.6
Education	−0.009	0.013	−16.0
Literate	0.009	0.012	17.0
Expenditure	0.003***	0.001	5.1
Roof	−0.001	0.001	−1.1
Floor	−0.000	0.001	−0.2
Toilet	−0.001***	0.000	−1.3
Lighting fuel	−0.000	0.000	−0.1
Cooking fuel	−0.000	0.001	−0.1
Clean water rainy	−0.002	0.004	−3.2
Clean water dry	−0.001	0.004	−2.5
Radio	0.024***	0.006	44.0
Mosquito net	0.002***	0.001	4.0
Illness	0.008***	0.002	14.0
**Due to difference in coefficients**			
Urban	−0.013	0.016	−23.2
Age	0.066	0.043	121.2
Married	0.001	0.006	0.6
Education	0.005	0.024	8.8
Literate	−0.010	0.023	−19.2
Expenditure	−0.046*	0.024	−85.0
Roof	0.048	0.001	88.2
Floor	−0.033	0.001	−60.5
Toilet	0.000**	0.000	−0.4
Lighting fuel	0.013	0.000	24.3
Cooking fuel	0.014	0.001	25.4
Clean water rainy	−0.010	0.050	−12.5
Clean water dry	0.002	0.042	4.2
Radio	0.002	0.008	3.7
Mosquito net	0.005	0.013	−8.5
Illness	0.003	0.010	5.3
Constant	−0.015	0.058	−27.7

Note: A detailed non-linear (logit) decomposition. The ‘Percent’ column gives the contribution of each variable to the overall difference in health status between men and women. This is computed by dividing the estimate by the overall difference (0.054). Total observations is 4104. *, **, ***. Statistically significant at the 10%, 5%, and 1% level, respectively.

#### Gender disparities in health status in rural areas

We do not find any significant gender disparities in SAH in urban areas (0.023, p > 0.10). As such, in the next subsection, we concentrate in rural areas only where the gender disparities in health are significantly large (0.099, p < 0.01). These results reveal that the gender health disparities are mostly pronounced in rural areas. Table 3 shows the econometric results of the decomposition exercise in rural areas only. First, we see that differences in observed characteristics (endowments) explain about 45% of the difference in health status between men and women in rural areas. The logit decomposition reveals that the gender gap is mostly explained by access to information, age, utilization of mosquito nets and clean toilets in rural Liberia. Access to information is found to narrow the difference in health status between the two groups by 29%. Access to current information and utilization of mosquito nets are found to reduce the gender gap between the two groups in the overall sample as well as in rural areas.

**Table 3.** Logit decomposition of men and women in health status in rural areas.

	Estimate	Standard error	Percent
Raw difference	0.099***	0.021	100
Explained: due to difference in characteristics	0.044***	0.01	44.9
Unexplained: due to difference in coefficients	0.055	0.026	55.1
**Due to difference in characteristics**			
Age	0.010***	0.001	9.8
Married	−0.001	0.011	−1.2
Education	−0.014	0.018	−14.3
Literate	0.020	0.016	20.0
Expenditure	−0.000	0.003	−0.2
Roof	0.001	0.001	−1.0
Floor	0.001	0.001	0.6
Toilet	0.001**	0.000	0.7
Lighting fuel	0.000	0.000	0.3
Cooking fuel	0.000	0.001	0.4
Clean water rainy	0.000	0.004	0.5
Clean water dry	−0.003	0.004	−2.8
Radio	0.028***	0.001	28.5
Mosquito net	0.001***	0.001	1.5
Illness	0.002	0.003	2.2
**Due to difference in coefficients**			
Age	0.099	0.062	99.7
Married	0.005	0.011	5.4
Education	0.019	0.024	19.0
Literate	−0.024	0.021	−24.5
Expenditure	−0.014	0.033	−14.2
Roof	0.050**	0.023	50.4
Floor	−0.033***	0.012	−32.8
Toilet	−0.000	0.010	−0.6
Lighting fuel	−0.010	0.020	−8.6
Cooking fuel	0.10	0.010	6.9
Clean water rainy	0.023	0.050	23.0
Clean water dry	−0.021	0.047	−20.7
Radio	0.010	0.010	9.8
Mosquito net	0.010	0.020	8.1
Illness	−0.010	0.014	−7.4
Constant	−0.059	0.081	−58.6

Note: A detailed non-linear (logit) decomposition. The ‘Percent’ column gives the contribution of each variable to the overall difference in health status between men and women. This is computed by dividing the estimate by the overall difference (0.098). Total observations is 2558. *, **, ***. Statistically significant at the 10%, 5%, and 1% level, respectively.

## Robustness checks

The robustness check model uses the same covariates for the overall sample (Table 2) with the exclusion of per-capita expenditure, and results are shown in Appendix 1. The gender disparities in health are significantly large and similar (0.054, p < 0.01), regardless of the exclusion of the wealth variable. The differences in endowments explain about 46% of the difference in health status between men and women. The logit decomposition reveals that the men–women gap is mostly explained by access to information, illness, age, toilet facility and utilization of mosquito nets. Access to information is the main contributor to the reduction in health inequality between the two groups, and it accounts for 45%. These results reveal that the model results are robust. The contribution of illness in explaining the gender differences in health measured in terms of self-assessed health is found to be 14%, which is quantitatively similar to previous results shown in Table 2.

## Discussion

To our knowledge, this is the first study decomposing health-related inequalities in self-assessed health status in Liberia. In our study, results reveal that a higher proportion of women reported illness in the last 30 days compared to men both in the overall and rural samples. Similar findings that women experience poorer health than men despite their longer life expectancy are also reported by [38,39]. There are some explanations why women have poorer health compared to men. Several studies argue that poorer health among women is due to biological as well as behavioural factors and cannot be explained by differences in socio-economic and demographic factors only [4,40–42]. Other studies highlight that women over-report worse health status than men [41–44]. Various authors have argued that women are more sensitive, interface with the healthcare system as caregivers of their children and easily report health conditions than men [43,44].

In our study, we found that the large gender disparities are confined to rural areas and absent in urban areas. The poor health status for rural women might be a reflection of inadequate health infrastructure and health care [45] as well as poor water and sanitation in rural areas of Liberia. Women in Liberia and other developing countries and in particular, in rural areas provide the bulk of manual labour in agriculture, yet they are already overburdened by child care and other household chores [46,47] and this has negative implications on their health. Hence, labour saving technologies in agriculture (e.g. threshers, dehullers, herbicides) and water extracting techniques (e.g. solar powered boreholes) should be promoted to reduce women labour burden and improve their health and well-being.

The non-linear decomposition results suggest that the gender inequalities in health are due to differences in household access to information, wealth and unequal utilization of mosquito nets. The main finding of our study is that these inequalities were mainly accounted for by gender inequalities in access to information, in particular listening to current news via the radio. This is possibly due to the increased access to information on health diets, malaria prevention as well as good health behaviours and treatment in radio owning households. Earlier studies found similar results that knowledge contributes significantly to the disparities in health [3,48]. For example [48], found that education, employment status, and depression contributed more to the gender gap observed in self-rated health and chronic diseases in South Korea.

We found that household expenditure (wealth) explained gender inequalities in self-assessed health status in Liberia, and this is compatible with the findings of previous studies in India [24] and Spain [38]. Higher levels of income and wealth tend to be associated with better health status, access to healthy food, health care and housing [17,18]. Given that wealth is correlated with health status, there is need for private and public sector in Liberia to create employment and income generating activities that absorb both men and women. Interventions that remove discrimination against women in health care, education, employment and income generating activities are crucial. The reduction of gender inequalities in these domains is also expected to improve women and children’s well-being, owing to women’s important role in food preparation and childcare [49]. Our findings confirm that utilization of mosquito nets is associated with a reduction in gender inequalities in health. The provision of insecticide-treated bed nets is one of the most important interventions of malaria control strategy [50,51] in Liberia. Although the country has made tremendous progress in malaria control [50], interventions that enhance wider coverage and increase consistent use of mosquito nets by people at risk of malaria should be promoted.

## Study limitations

The limitations of this study relate to the fact that both the dependent and independent variables are self-reported and are likely to have reporting bias and recall lapse. The data used are cross-sectional and use Oaxaca-Blinder decomposition for analysis and, therefore, we cannot establish any causality between self-assessed health and different socioeconomic, wealth and health-related variables. Despite these limitations, this study is important in that it gives an understanding and quantification of the drivers and magnitude of gender inequalities in health status.

## Conclusion

The study revealed substantial gender health inequalities, characterised by women disadvantages. However, inferences from these findings should be treated with caution. As discussed above, this is an observational study and one should not attribute causality to these findings. Mindful of this important shortcoming, these findings have potentially some important implications for policy. First, carefully designed health information programmes (e.g. through electronic and print media) could generate positive outcomes in health, as radio listening is associated with a reduction in the women-men gap in self-reported health status. Second, interventions and policies that improve wealth (for example employment and income generation activities) need to be reinforced as they contribute significantly to reduction of inequality. Given that the majority of poor people reside in rural areas and women have poor health status, there is need for pro-poor and gender responsive interventions. The health sector should ensure that all people, regardless of gender have improved access to quality mosquito nets and health services in-order to reduce the disease burden. Third, efforts to expand access to improved water and sanitation and clean fuel energy (e.g. clean water access and renewable energy) in rural areas as well as among women sub-populations should be expanded to improve health. Within the context of Liberia, the paper argues for an increase in government, private sector and civil society’s role in increasing access to health information, wealth creation interventions and quality mosquito nets in order to redress gender health inequalities. Finally, we recommend, further work in the form of rigorous impact evaluations of interventions in these areas to confirm the validity of these ideas.

## Paper context

While many studies have documented the gender disparities in health, few have evaluated the magnitude and drivers of gender inequalities. This paper quantifies the magnitude and drivers of gender inequalities in health using decomposition technique. We document substantial gender inequalities in health status, characterised by women disadvantages. Inequalities are mainly explained by the distribution of endowments. Programmes that equalise access to information, wealth creation interventions and mosquito nets would reduce the gender gaps in health.

## Appendix Appendix 1. Logit decomposition of men-women gap in health status in overall sample

**Table:**

	Estimate	Standard error	Percent
Raw difference	0.054***	0.016	100
Explained: due to difference in characteristics	0.025***	0.012	46.4
Unexplained: due to difference in coefficients	0.029	0.020	53.6
**Due to difference in characteristics**			
Urban	−0.001	0.003	−1.5
Age	−0.004***	0.000	−8.5
Married	0.001	0.009	1.7
Education	−0.009	0.013	−16.0
Literate	0.010	0.012	18.4
Roof	−0.000	0.002	−0.9
Floor	−0.000	0.000	−0.5
Toilet	−0.001***	0.002	−1.4
Lighting fuel	0.000	0.000	0.1
Cooking fuel	−0.001	0.002	−1.8
Clean water rainy	−0.001	0.004	−2.9
Clean water dry	−0.002	0.004	−2.6
Radio	0.024***	0.006	44.9
Mosquito net	0.002***	0.006	3.9
Illness	0.007***	0.002	13.5
**Due to difference in coefficients**			
Urban	−0.014	0.016	−25.8
Age	0.060	0.045	111.3
Married	−0.000	0.006	−0.6
Education	0.007	0.026	12.8
Literate	−0.017	0.024	−31.1
Roof	0.046	0.026	85.0
Floor	−0.036	0.017	−65.8
Toilet	0.000	0.008	0.1
Lighting fuel	0.012	0.017	21.3
Cooking fuel	0.008	0.014	15.1
Clean water rainy	−0.004	0.047	−8.1
Clean water dry	0.001	0.044	−2.0
Radio	−0.001	0.008	−1.0
Mosquito net	−0.003	0.013	−6.1
Illness	0.003	0.011	5.1
Constant	−0.033	0.059	−60.7

Note: A detailed non-linear (logit) decomposition. The ‘Percent’ column gives the contribution of each variable to the overall difference in health status between men and women. This is computed by dividing the estimate by the overall difference (0.054). Total observations is 4104. *, **, ***. Statistically significant at the 10%, 5%, and 1% level, respectively.

## References

[1] Pollard TM, Hyatt SB. Sex, gender and health: integrating biological and social perspectives In: Pollard TM, Hyatt SB, editors. Sex, gender, and health. UK: Cambridge University Press. 1999: 1–10.

[2] WHO Gender and health [Internet]. [cited 2018 9 24]. Available from: http://www.who.int/news-room/fact-sheets/detail/gender

[3] Ataguba JE-O, Day C, Di McIntyre Explaining the role of the social determinants of health on health inequality in South Africa. Glob Health Action. 2015;8:28865. 26385543 10.3402/gha.v8.28865 PMC4575416

[4] Bora JK, Saikia N Gender differentials in self-rated health and self-reported disability among adults in India. PLoS ONE. 2015;10:e0141953. 26536133 10.1371/journal.pone.0141953 PMC4633186

[5] Simon JG, Boer JBD, Joung IMA, et al How is your health in general? A qualitative study on self-assessed health. Eur J Public Health. 2005;15:200–208. 15941763 10.1093/eurpub/cki102

[6] Blomstedt Y, Souares A, Niamba L, et al Measuring self-reported health in low-income countries: piloting three instruments in semi-rural Burkina Faso. Glob Health Action. 2012;5 DOI:10.3402/gha.v5i0.8488 PMC3404415 22833712

[7] Idler EL Survival, functional limitations, and self-rated health in the NHANES I epidemiologic follow-up study, 1992. Amer J Epidem. 2000;152:874–883. 10.1093/aje/152.9.874 11085400

[8] Duboz P, Boëtsch G, Gueye L, et al Self-rated health in Senegal: A comparison between urban and rural areas. PLoS ONE. 2017;12:e0184416. 28886107 10.1371/journal.pone.0184416 PMC5590920

[9] Onadja Y, Atchessi N, Soura BA, et al Gender differences in cognitive impairment and mobility disability in old age: a cross-sectional study in Ouagadougou, Burkina Faso. Arch Gerontol Geriatr. 2013;57:311–318. 23827740 10.1016/j.archger.2013.06.007

[10] Singh PK, Jasilionis D, Oksuzyan A Gender difference in cognitive health among older Indian adults: A cross-sectional multilevel analysis. SSM Popul Health. 2018;5:180–187. 30073185 10.1016/j.ssmph.2018.06.008 PMC6068074

[11] Etherington N Re-evaluating gender differences in self-rated health: the importance of cohort. J Women Aging. 2017;29:150–162. 27441464 10.1080/08952841.2016.1108737

[12] Rohlfsen LS, Jacobs Kronenfeld J Gender differences in trajectories of self-rated health in middle and old age: an examination of differential exposure and differential vulnerability. J Aging Health. 2014;26:637–662. 24700604 10.1177/0898264314527477

[13] Miszkurka M, Zunzunegui MV, Langlois EV, et al Gender differences in mobility disability during young, middle and older age in West African adults. Glob Public Health. 2012;7:495–508. 22085342 10.1080/17441692.2011.630676

[14] Björkman-Nyqvist M Income shocks and gender gaps in education: evidence from Uganda. J Deve Econ. 2013;105:237–253.

[15] Olsen RN, Coppin A The determinants of gender differences in income in Trinidad and Tobago. J Deve Stud. 2001;37:31–56.

[16] Dewalt DA, Berkman ND, Sheridan S, et al Literacy and health outcomes: a systematic review of the literature. J Gen Intern Med. 2004;19:1228–1239. 15610334 10.1111/j.1525-1497.2004.40153.x PMC1492599

[17] Subramanian SV, Neve J-WD Social determinants of health and the international monetary fund. Proc Natl Acad Sci U S A. 2017;114:6421–6423. 28600352 10.1073/pnas.1706988114 PMC5488961

[18] Hernandez LM, Blazer DG Genes, behavior, and the social environment: moving beyond the naturenurture debate. Washington DC: National Academies Press; 2006. 20669442

[19] Amegah AK, Quansah R, Jaakkola JJK Household air pollution from solid fuel use and risk of adverse pregnancy outcomes: a systematic review and meta-analysis of the empirical evidence. PLoS ONE. 2014;9:e113920. 25463771 10.1371/journal.pone.0113920 PMC4252082

[20] LISGIS Liberia demographic and health survey. Monrovia: Liberia; 2013.

[21] UNICEF At a glance: Liberia [Internet]. [cited 2018 9 21]. Available from: https://www.unicef.org/infobycountry/liberia_2513.html

[22] Gustafsson PE, Sebastián MS, Mosquera PA Meddling with middle modalities: a decomposition approach to mental health inequalities between intersectional gender and economic middle groups in northern Sweden. Glob Health Action. 2016;9:32819. 27887668 10.3402/gha.v9.32819 PMC5124119

[23] Amroussia N, Gustafsson PE, Mosquera PA Explaining mental health inequalities in Northern Sweden: a decomposition analysis. Glob Health Action. 2017;10:1305814. 28562191 10.1080/16549716.2017.1305814 PMC5496092

[24] Pandey A, Ladusingh L Socioeconomic correlates of gender differential in poor health status among older adults in India. J Applied Geront. 2013;34:879–905. 10.1177/0733464813481850 24652876

[25] Sia D, Onadja Y, Nandi A, et al What lies behind gender inequalities in HIV/AIDS in sub-Saharan African countries: evidence from Kenya, Lesotho and Tanzania. Health Pol Plan. 2014;29:938–949. 10.1093/heapol/czt075 PMC4186212 24345343

[26] Patra S Socio-cultural correlates and risky sexual behaviour influencing prevalence of HIV/AIDS and STIs in Uganda: A gender perspective. Cogent Soc Scien. 2016;2:1757.

[27] Powers DA, Yoshioka H, Yun M-S mvdcmp: multivariate decomposition for nonlinear response models. Stata J. 2011;11:556–576.

[28] Oaxaca R Male-female wage differentials in urban labor markets. Intern Econ Rev. 1973;14:693–709.

[29] Parnarouskis L, Stevenson A, Lange BCL, et al The impact of transactional sex with teachers on public school students in Monrovia, Liberia - a brief report. Vulnerable Child Youth Stud. 2017;12:328–333. 29354188 10.1080/17450128.2017.1300721 PMC5773053

[30] Sobkoviak RM, Yount KM, Halim N Domestic violence and child nutrition in Liberia. Soc Sci Med. 2012;74:103–111. 22185910 10.1016/j.socscimed.2011.10.024

[31] World Bank Liberia - household income and expenditure survey 2014-2015. Washington DC: USA; 2018 5.

[32] Angoua ELE, Dongo K, Templeton MR, et al Barriers to access improved water and sanitation in poor peri-urban settlements of Abidjan, Côte d‘Ivoire. PLoS ONE. 2018;13:e0202928. 30153297 10.1371/journal.pone.0202928 PMC6112649

[33] Filmer D, Pritchett LH Estimating wealth effects without expenditure data—or tears: an application to educational enrollments in states of India. Demography. 2001;38:115–132. 11227840 10.1353/dem.2001.0003

[34] Agusto FB, Del Valle SY, Blayneh KW, et al The impact of bed-net use on malaria prevalence. J Theor Biol. 2013;320:58–65. 23246718 10.1016/j.jtbi.2012.12.007 PMC3623563

[35] Fairlie RW An extension of the Blinder-Oaxaca decomposition technique to logit and probit models. J Econ Soc Meas. 2005;30:305–316.

[36] Yun M-S A simple solution to the identification problem in detailed wage decompositions. Econ Inq. 2005;43:766–772.

[37] Fiedler JL, Lividini K, Bermudez OI, et al Household Consumption and Expenditures Surveys (HCES): a primer for food and nutrition analysts in low- and middle-income countries. Food Nutr Bull. 2012;33:S170–S184. 23193768 10.1177/15648265120333S205

[38] Malmusi D, Vives A, Benach J, et al Gender inequalities in health: exploring the contribution of living conditions in the intersection of social class. Glob Health Action. 2014;7:23189. 24560257 10.3402/gha.v7.23189 PMC3927744

[39] Zunzunegui M-V, Alvarado B-E, Béland F, et al Explaining health differences between men and women in later life: a cross-city comparison in Latin America and the Caribbean. Soc Sci Med. 2009;68:235–242. 19036488 10.1016/j.socscimed.2008.10.031

[40] Crimmins EM, Shim H, Zhang YS, et al Differences between men and women in mortality and the health dimensions of the morbidity process. Clin Chem. 2019;65:135–145. 30478135 10.1373/clinchem.2018.288332 PMC6345642

[41] Regitz-Zagrosek V Sex and gender differences in health. Science & society series on sex and science. EMBO Rep. 2012;13:596–603. 22699937 10.1038/embor.2012.87 PMC3388783

[42] Rieker PP, Bird CE Rethinking gender differences in health: why we need to integrate social and biological perspectives. J Gerontol B. 2005;60:S40–S47. 10.1093/geronb/60.special_issue_2.s40 16251589

[43] Wandera SO, Golaz V, Kwagala B, et al Factors associated with self-reported ill health among older Ugandans: a cross sectional study. Arch Gerontol Geriatr. 2015;61:231–239. 26043957 10.1016/j.archger.2015.05.006 PMC4534344

[44] Kabir ZN, Tishelman C, Agüero-Torres H, et al Gender and rural–urban differences in reported health status by older people in Bangladesh. Arch Gerontol Geriatr. 2003;37:77–91. 12849075 10.1016/s0167-4943(03)00019-0

[45] Stanturf JA, Goodrick SL, Warren ML, et al Social vulnerability and ebola virus disease in rural Liberia. PLoS ONE. 2015;10:e0137208. 26325519 10.1371/journal.pone.0137208 PMC4556488

[46] Adjei NK, Brand T, Zeeb H Gender inequality in self-reported health among the elderly in contemporary welfare countries: A cross-country analysis of time use activities, socioeconomic positions and family characteristics. PLoS ONE. 2017;12:e0184676. 28949984 10.1371/journal.pone.0184676 PMC5614435

[47] Graham JP, Hirai M, Kim-S-S An analysis of water collection labor among women and children in 24 Sub-Saharan African Countries. PLoS ONE. 2016;11:e0155981. 27248494 10.1371/journal.pone.0155981 PMC4889070

[48] Chun H, Khang Y-H, Kim I-H, et al Explaining gender differences in ill-health in South Korea: the roles of socio-structural, psychosocial, and behavioral factors. Soc Sci Med. 2008;67:988–1001. 18632197 10.1016/j.socscimed.2008.05.034

[49] Malapit HJL, Quisumbing AR What dimensions of women’s empowerment in agriculture matter for nutrition in Ghana? Food Policy. 2015;52:54–63.

[50] Bawo LL, Harries AD, Reid T, et al Coverage and use of insecticide-treated bed nets in households with children aged under five years in Liberia. Public Health Action. 2012;2:112–116. 26392967 10.5588/pha.12.0040 PMC4463064

[51] Noor AM, Mutheu JJ, Tatem AJ, et al Insecticide-treated net coverage in Africa: mapping progress in 2000–07. Lancet. 2009;373:58–67. 19019422 10.1016/S0140-6736(08)61596-2 PMC2652031

